# Exploring drought tolerance in wild and traditional olive varieties from the Southern Levant

**DOI:** 10.3389/fpls.2025.1547174

**Published:** 2025-02-12

**Authors:** Basappa Adi, Arnon Dag, Elad Ben-Dor, Gilad Gabay, Oz Barazani

**Affiliations:** ^1^ French Associates Institute for Agriculture and Biotechnology of Drylands, The Jacob Blaustein Institutes for Desert Research, Ben-Gurion University of the Negev, Sede-Boker, Israel; ^2^ Department of Fruit Tree Sciences, Gilat Research Center, Agricultural Research Organization, Volcani Institute, Gilat, Israel; ^3^ Department of Vegetable and Field Crops, Institute of Plant Sciences, Agricultural Research Organization, Rishon LeZion, Israel

**Keywords:** adaptation, climate change, drought, olive, traditional cultivars, wild olives

## Abstract

Local olive germplasm of the southern Levant includes wild populations of var. *sylvestris* and local traditional cultivars that are thought to be well-adapted to the region’s arid conditions. By controlling water availability, we tested the response of the Barnea cultivar, two local traditional cultivars (MLL1 and MLL7) and var. *sylvestris* to low (100%), moderate (33%), and severe (10%) evapotranspiration (ETa) conditions. Measurements of stomatal conductance, relative water content, stem water potential, and the net photosynthesis showed a stronger response of the Barnea cultivar to reduced ETa conditions in comparison to the other three investigated groups. Additionally, when exposed to 100% ETa, the net photosynthesis capacity of MLL1 was significantly higher than that measured in MLL7. Therefore, net photosynthesis, as an indicator of tree productivity, can explain the dominance of MLL1 (Souri cultivar) in local traditional orchards and the negligible abundance of MLL7 (unknown cultivar) as a fruit-bearing tree. Considering that climate change is already influencing olive cultivation, the results of this study stress the potential of the southern Levant local olive germplasm in maintaining sustainable olive horticulture.

## 1 Introduction

**Figure 1:**
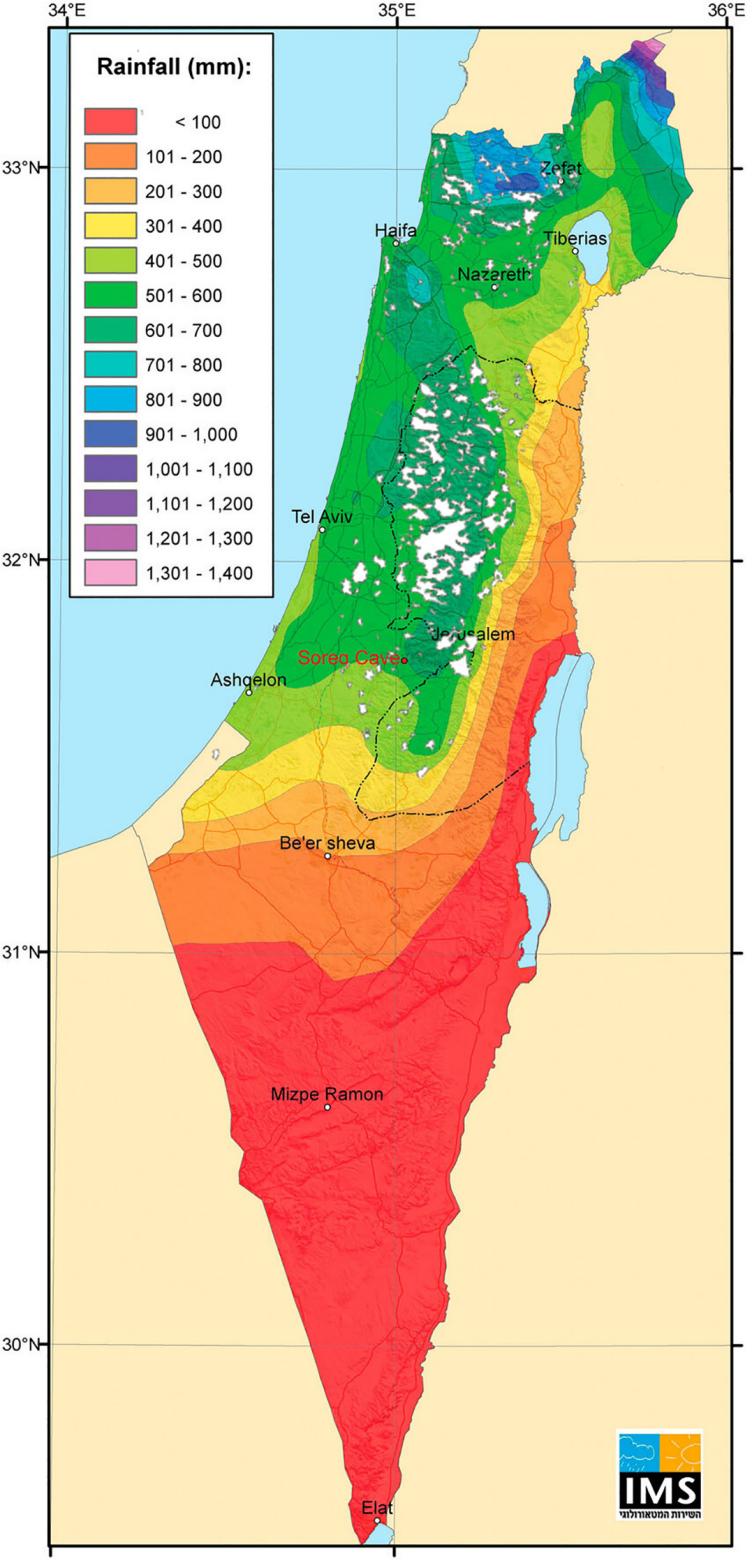
The spread of traditional olive groves in the Southern Levant. The map illustrates the distribution of olive orchards in 1935 (Rafael Frankel et al., 1994), overlaid with the region’s annual participation gradient (data source: the Israel Meteorological Service).

Since antiquity, olive oil and table olives have been major components of the daily diet and culture of people in the Mediterranean, specifically in the eastern Mediterranean (Galili et al., 2021; Barazani et al., 2023). Thus, for thousands of years, olive orchards have been a major feature of the rural Mediterranean landscape. Data from the Food and Agriculture Organization of the United Nations (FAO) (https://www.fao.org/faostat/en/) indicates that there are currently 9.4 million hectares of olive orchards worldwide, with 90% concentrated in the Mediterranean Basin. In the southern Levant (modern Israel and the Palestinian Authority), most of the olive cultivation area is still based on traditional agriculture practices (92.5% of a total of 120,000 ha) (Dag et al., 2024). Traditional olive horticulture in this region, based on local cultivars growing under rain-fed conditions, is mostly limited to temperate semi-arid zones (≥350 mm annual rainfall) (**Figure 1**). Nevertheless, rain-fed orchards are expanding into more arid zones, and individual olive trees, along with relicts of ancient abandoned orchards, are scattered throughout the region and even in the Negev desert (Ashkenazi et al., 2018; Tepper et al., 2022). Moreover, new irrigation and fertilization agro-techniques have expanded olive cultivation zone into more arid regions (Dag et al., 2004).

Using multi-locus linage analysis (MLL) of 14 microsatellite loci, Barazani et al. (2014) detected two main local cultivars among more than 300 old living trees: MLL1 and MLL7. The widespread abundance of MLL1 as a fruit-bearing tree confirmed its identification as the common Souri cultivar. In contrast, the nomenclature of MLL7 is still unknown (Barazani et al., 2014). Genetic analysis also indicated that the majority of rootstocks (55%) of old olive trees originated from sexually reproduced saplings (Barazani et al., 2014), most likely collected in the wild from *Olea europaea* subsp. *europaea* var. *sylvestris* (Barazani et al., 2016).

Several populations of var. *sylvestris*, the wild ancestor of cultivated olives, are still spread around the Mediterranean Basin (Barazani et al., 2016; Tourvas et al., 2023; Ben-Dor et al., 2024; Zunino et al., 2024). Moreover, studies have shown the utilization of var. *sylvestris* as a rootstock of grafted old olives in the southern Levant (Barazani et al., 2014, 2016), north-western Africa (Ater et al., 2016; Aumeeruddy-Thomas et al., 2017), and the Iberian Penisula (Díez et al., 2011). The genetic variation in wild populations and traditional cultivars is considered a valuable source of adaptive traits (Hannachi et al., 2009; Barazani et al., 2023, 2024). Thus, hypothesizing that local olive germplasm evolved to withstand the southern Levantine semi-arid conditions (Barazani et al., 2008), we aimed to test the response of MLL1 (Souri), MLL7, and local var. *sylvestris* to drought stress under controlled irrigation conditions.

## 2 Materials and methods

Stem cuttings, 5 mm in diameter, were taken from trees of MLL1 and MLL7 growing in the Gilat germplasm collection (Barazani et al., 2023), and from three individuals of *O. europaea* subsp. *europaea* var. *sylvestris* growing naturally in Atlit (Ben-Dor et al., 2024). Stem cuttings were also collected from the Barnea cultivar (Gilat germplasm), to represent a local modern variety adapted for intensive irrigation practices (Lavee, 2009).

The cuttings were rooted under mist conditions as previously described (Dag et al., 2012) and then grown in 3 L pots with commercial potting media composed of coconut fibers, peat, and tuff (Deshanit, Israel). Pots were organized in a block design including 12 pots of each of the four investigated varieties. Water content was decreased to 33% and 10% ETa (actual evapotranspiration), which represents the sum of evaporation from the soil and plant surface along with transpiration. Daily measurement of pot weight were used to calculate daily ETa and subsequently control 33% and 10% ETa in each pot by irrigation. Each treatment group included four plants of each variety (MLL1, MLL7, Barnea, and var. *sylvestris*). The ETa treatments were maintained for three weeks during which physiological parameters were measured: Stem water potential (SWP) was measured at midday using a Scholander-type pressure chamber (MRC, Israel), and the leaf relative water content (RWC) was determined following Yamasaki and Dillenburg (1999). Measurements of net photosynthesis (µmol CO_2_ m^−2^ s^−1^) and stomatal conductance (mmole H_2_O m^−2^ s^−1^) were performed between 9:00 and 11:00 a.m. using a Licor 6400XT device (LI-COR Inc., Lincoln, NE, USA), keeping light intensity at 1000 µmol photon m^−2^ s^−1^ and ambient CO_2_ concentration at 400 µmole CO_2_ mol^−1^ air. Stomatal conductance was measured four times every 7 days during the course of the experiment, while net photosynthesis, SWP and RWC were determined 21 days after exposure to the different irrigation treatments.

Results in the figures are presented as mean ± standard errors (SE), and *post-hoc* statistical tests were conducted with JMP Pro 16.0.0 (SAS Institute Inc.).

## 3 Results and discussion

**Figure 2:**
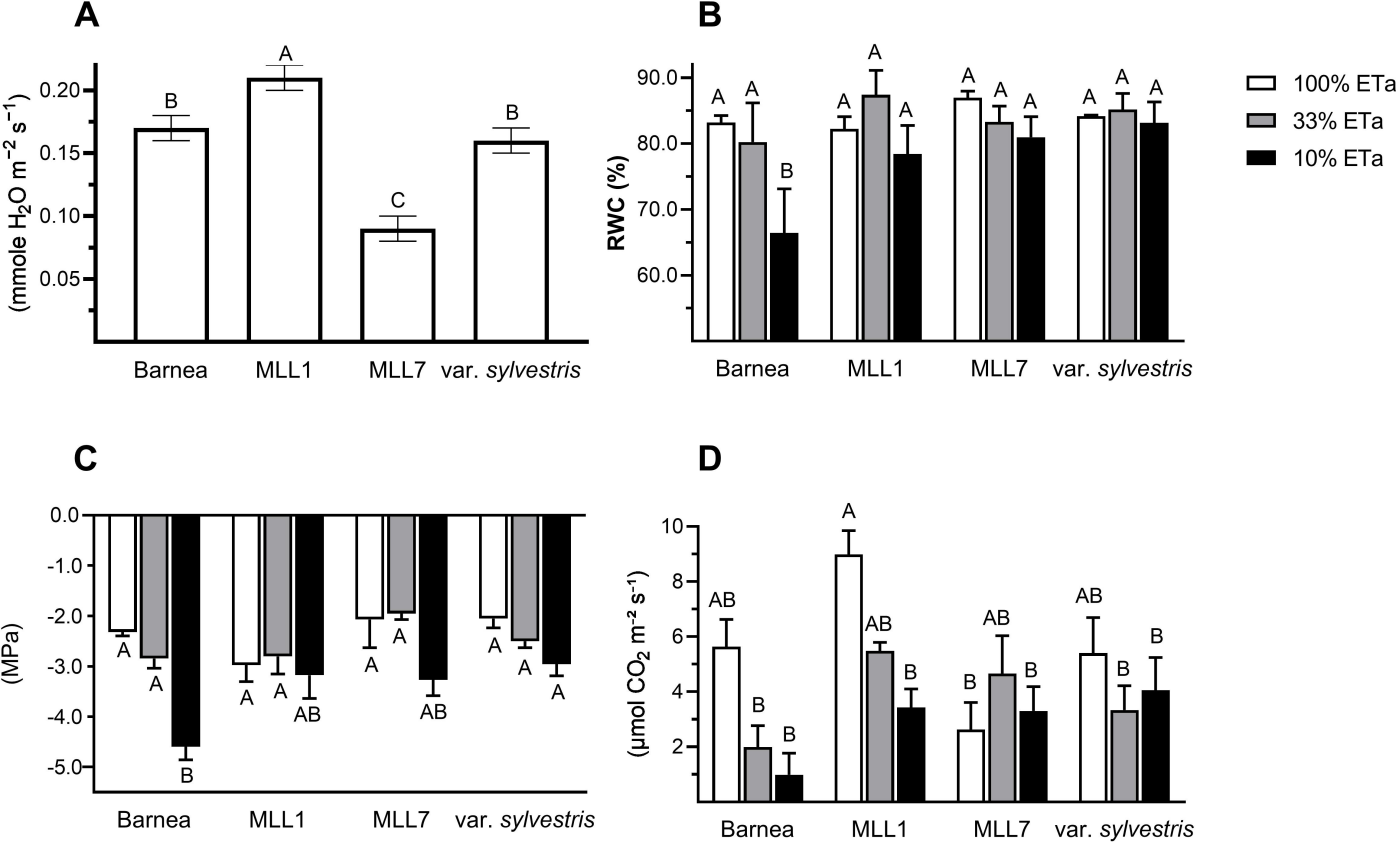
The influence of moderate (33% ETa) and severe drought stress (10% ETa) on physiological parameters, compared to 100% ETa. Results of the stomatal conductance summarizing the measurements that were taken in olives growing in 100% ETa conditions at various time points throughout the experiment **(A)**. Measurements of relative water content **(B)**, stem water potential **(C)**, and net photosynthesis **(D)** were taken three weeks after the commencement of the drought treatment. Different letters above bars indicate statistically significant differences between cultivars and treatments (ANOVA Tukey’s HSD).

**Figure 3:**
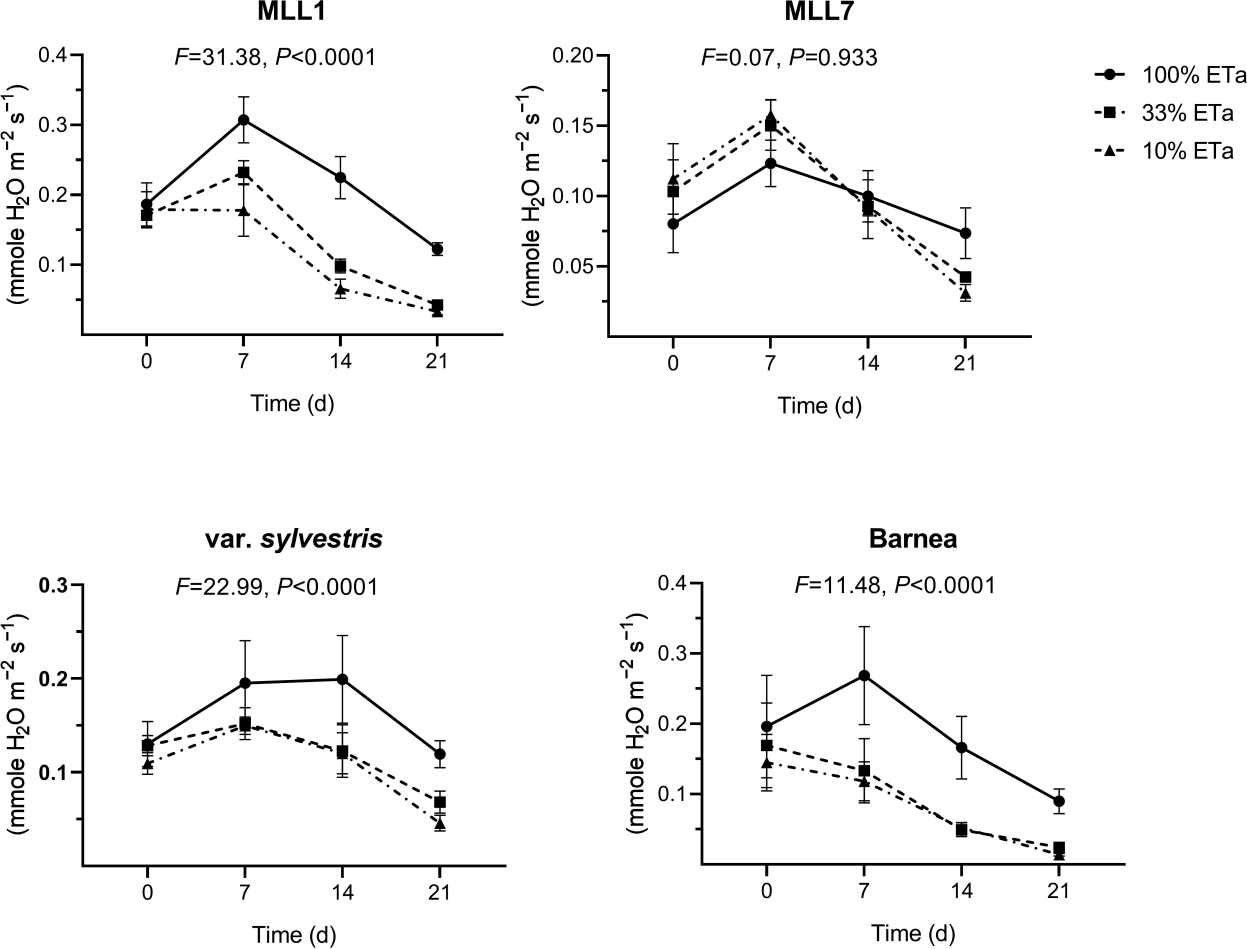
The influence of moderate (33% ETa) and severe drought stress (10% ETa) on stomatal conductance in the four investigated olive varieties. Measurements were conducted at the beginning of the experiments and at three subsequent time points. Results of the ANOVA with repeated measures, assessing the interaction between treatment and time, are presented above each graph.

In general, stomatal conductance measured in one-year old olive trees growing under 100% ETa was about two times significantly lower in MLL7 (0.18 ± 0.01 mmole H_2_O m^−2^ s^−1^) than in the other three investigated varieties (**Figure 2A**). The two low ETa treatments had a significant influence on MLL1 stomatal conductance, as was the case for Barnea and *O. europaea* subsp. *europaea* var. *sylvestris*, but not in MLL7 (ANOVA with repeated measures) (**Figure 3**). In MLL7, a significant 3.7-fold reduction in stomatal conductance, compared to 100% ETa, was only evident 21 days after exposure to severe drought (10% ETa) (**Figure 3**). In contrast, in the Barnea cultivar and MLL1, a significant reduction in stomatal conductance was measured already seven days after exposure to 33% ETa (Student’s *t*-test, *P*<0.05), and in *O. europaea* subsp. *europaea* var. *sylvestris*, a significant reduction in stomatal conductance was measured 14 days after exposure to both of the drought treatments (**Figure 3**). Additionally, MLL1, MLL7 and *O. europaea* subsp. *europaea* var. *sylvestris* maintained similar RWC (**Figure 2B**) and SWP (**Figure 2C**) in the three ETa treatments. However, in the Barnea cultivar, when exposed to 10% ETa, a significant 1.3- and 2.0-fold reduction was measured in RWC and SWP at the end of the experiment, respectively (ANOVA Tukey HSD, *P*<0.05) (**Figures 2B, C**). These results support previous findings showing the sensitivity of the Barnea cultivar to drought (Tugendhaft et al., 2016; Barzilai et al., 2021).

Altogether, the ability of MLL7 to maintain similar low stomatal conductance, WRC, SWP, and net photosynthesis across all ETa treatments, and throughout the experiment (**Figures 2**, **3**), suggests that its physiological response to water stress is less pronounced than in MLL1, Barnea and *O. europaea* subsp. *europaea* var. *sylvestris*. Thus, our results suggest that past selection processes favored cultivars well adapted to local conditions. The drought resilience of MLL7 may also explain the prominence of MLL7_rootstock_/MLL1_scion_ combination among ancient living olive trees in the Southern Levant (Barazani et al., 2014, 2017). Supporting its resilience to harsh arid environments, MLL7 survived as a fruit-bearing tree in an agricultural plot in the Negev desert (<50 mm annual rainfall year^-1^) for at least 500 years without any irrigation (Tepper et al., 2022). However, the low photosynthesis capacity in MLL7 growing in 100% ETa was significantly 3.4 times lower than that measured in MLL1 (**Figure 2D**). Low photosynthetic capacity is associated with reduced productivity and lower yields (Zhu et al., 2010), which might explain the negligible abundance of MLL7 as a fruit-bearing tree compared to MLL1 (Barazani et al., 2014).

Grafting is considered to increase the survival and growth of propagated olives (Foxhall, 2007), especially of cultivars that do not root easily (Barazani et al., 2014). The utilization of specific clones as rootstocks was reported by Zohary et al. (2012) as a common propagation technique used by traditional olive growers in Turkey. Additionally, it has been shown that grafting the Picual cultivar (scion) on rootstocks of *O. europaea* subsp. *europaea* var. *sylvestris* improved tree vigor (Díaz-Rueda et al., 2020). Although our experiment did not include grafted olives, the findings support the resilience of *O. europaea* subsp. *europaea* var. *sylvestris* to Mediterranean abiotic stress conditions (e.g., Ozturk et al., 2010; Kassout et al., 2021, 2024; Tadić et al., 2024). The role of saplings (segregating populations) as rootstocks in promoting fruit tree vigor is well-known (Warschefsky et al., 2016). In olives, grafting of the cultivar Coratina on various rootstocks emphasized the importance of grafting in ensuring the sustainability of olives under drought conditions (El Said et al., 2024). Accordingly, further comparative common garden experiments with grafted olives, utilizing both southern Levant local cultivars and var. *sylvestris* as rootstocks and scions (e.g., var. *sylvestris*_rootstock_/MLL1_scion_, MLL7_rootstock_/Barnea_scion_, var. *sylvestris*_rootstock_/Barnea_scion_), are needed to examine the potential advantage of this agronomic technique and local germplasm in controlling tree vigor under abiotic stress conditions.

Although the olive tree is considered well-adapted to Mediterranean environments, a decrease in its resilience to changing environmental conditions has already been reported (Fraga et al., 2020; Kaniewski et al., 2023). Assuming that the rootstocks of ancient grafted olive trees are, and have been, essential for survival and resilience over centuries in arid and semi-arid conditions in the region, the overall results of this study underscore the agronomic potential of local germplasm in agriculture and breeding programs, ensuring olive horticulture better suited for the changing environments.

## Data Availability

The original contributions presented in the study are included in the article/supplementary material. Further inquiries can be directed to the corresponding authors.
